# Energy and Delay Aware Data Aggregation in Routing Protocol for Internet of Things

**DOI:** 10.3390/s19245486

**Published:** 2019-12-12

**Authors:** Sankar Sennan, Sathiyabhama Balasubramaniyam, Ashish Kr. Luhach, Somula Ramasubbareddy, Naveen Chilamkurti, Yunyoung Nam

**Affiliations:** 1 Department of Computer Science and Engineering, Sona College of Technology, Salem 636005, India; sankar.cse@sonatech.ac.in (S.S.); sathiyabhama@sonatech.ac.in (S.B.); 2 Department of Electrical and Communication Engineering, The PNG University of Technology, Lae 411, Papua New Guinea; ashishluhach@gmail.com; 3 Department of Information Technology, VNR Vignana Jyothi Institute of Engineering &Technology, Hyderabad 500090, India; svramasubbareddy1219@gmail.com; 4 Department of Computer Science and IT, La Trobe University, Melbourne 3086, Australia; N.Chilamkurti@latrobe.edu.au; 5 Department of Computer Science and Engineering, Soonchunhyang University, Asan31538, Korea

**Keywords:** Internet of Things, data aggregation, compressed sensing theory, residual energy

## Abstract

Energy conservation is one of the most critical problems in Internet of Things (IoT). It can be achieved in several ways, one of which is to select the optimal route for data transfer. IPv6 Routing Protocol for Low Power and Lossy Networks (RPL) is a standardized routing protocol for IoT. The RPL changes its path frequently while transmitting the data from source to the destination, due to high data traffic in dense networks. Hence, it creates data traffic across the nodes in the networks. To solve this issue, we propose Energy and Delay Aware Data aggregation in Routing Protocol (EDADA-RPL) for IoT. It has two processes, namely parent selection and data aggregation. The process of parent selection uses routing metric residual energy (RER) to choose the best possible parent for data transmission. The data aggregation process uses the compressed sensing (CS) theory in the parent node to combine data packets from the child nodes. Finally, the aggregated data transmits from a downward parent to the sink. The sink node collects all the aggregated data and it performs the reconstruction operation to get the original data of the participant node. The simulation is carried out using the Contiki COOJA simulator. EDADA-RPL’s performance is compared to RPL and LA-RPL. The EDADA-RPL offers good performance in terms of network lifetime, delay, and packet delivery ratio.

## 1. Introduction

IoT is an emerging technology in Information and Technology (IT) [1]. It is a collection of internet-connected embedded devices that are capable of sensing and transmitting data from one location to another location without human support [2]. The name IoT indicates that things are connected tothe internet via communication technologies such as wireless sensor networks, near field communication, radio frequency identification, and Bluetooth [3]. IoT provides a lot of potential benefits in all aspects of human life [4]. IoT applications include smart homes, industrial Internet, smart supply chains, smart cities, wearables, smart grids, connected health, connected cars, smart farming, smart retail, etc. [5].

LLN is a type of IoT network which consists of routers and nodes restricted by resources [6]. The Routing Protocol for Low Power and Lossy Networks (RPL) has been standardized by IETF [7]. RPL forms the Destination Oriented Directed Acyclic Graph (DODAG) to transfer the data from the source to the Sink. The DODAG consists of a DODAG root and DODAG nodes. The top of a DODAG node is called DODAG’s root and the rest of the nodes are called DODAG nodes. The direction of the participant node towards the root of DODAG is called up routing, and vice versa is called down routing [8]. It supports traffic patterns, including point to multipoint, multipoint to point, and point to point [9]. The participant node chooses the best parent based on the objective function, which can decide on the application requirements [10].

Energy is a scarce resource in IoT. Therefore, energy conservation is a significant challenge in IoT [11]. Many techniques are proposed to reduce energy consumption in IoT. Energy-aware routing, sleeping mechanisms, and congestion-aware routing and data aggregation are the available techniques in IoT. In energy-aware routing, the existing protocols are energy-aware solar routing, node reliance techniques, multi-hop hierarchical clustering, duty cycling, and data-driven approaches to extend the network lifetime [12].

Data aggregation is a process of collecting the data and aggregates it from the sensor’s node. It is one of the essential processes to remove redundant data and save energy [13]. The primary objective of the data aggregation is to collect and aggregate the data. Also, it can be extended the network lifetime [14]. These techniques are suitable for some applications like temperature monitoring and gas leakage monitoring [15]. The existing data aggregation techniques are clustering and region-based routing to reduce duplicate data transmission across the network [16].

In IoT Routing, we need a suitable technique to aggregate the data among the network nodes. Hence, the Compressed Sensing (CS) theory is adopted in IoT, which is widely used in Wireless Sensor Networks (WSN) [17]. CS is a new theory that provides compression, coding, and decoding to reduce storage [18]. The applications for CS theory include data networks, digital images and video cameras, sensor networks, etc. CS is a beneficial technique to improve the performance of IoT. Also, it can be used for signal detection and processing, channel estimation, etc. [19].

The contribution of the proposed EDADA-RPL is to distribute the data and perform the single-hop data aggregation. It has two processes, namely parent selection and data aggregation. The process of parent selection uses routing metric residual energy (RER) to choose the best possible parent for data transmission. The data aggregation process uses the compressed sensing (CS) theory in each parent node to aggregate the data packets from the child nodes. Finally, the aggregated data transmits from a downward parent to the DODAG root.

The primary highlights of the proposed Energy and Delay Aware Data Aggregation in RPL (EDADA-RPL) are the following:

- The EDADA-RPL’s primary goal is to prevent redundant data transmission from the source to the root of DODAG.
- The CS theory-based data aggregation avoids redundant data transmission and prolongs the network lifetime.
- The efficiency of EDADA -RPL is assessed using COOJA simulator.

The paper is organized in the following way: the related work is described in Section 2. The network model is represented in Section 3. Section 4 discusses the CS theory. Section 5 represents the Energy and Delay Aware Data Aggregation in RPL. Section 6 discusses the results and discussion. Section 7 is the conclusion of the paper.

## 2. Related Works

In this section, we discuss energy-aware data aggregation techniques in RPL to increase the packet delivery ratio, decrease the delay, and improve the lifetime of the network in IoT.

Mohammad Hossein Homaei et al. [20] proposed an enhanced data aggregation method for IoT. It proposed a distributed method to balance the child node and reduce the congestion among the nodes in the network. Also, a learning automata-based dynamic data aggregation technique is proposed to aggregate the data in RPL (LA-RPL). Each node has a learning automaton to perform the data aggregation and transmission from one node to another node. The simulation has been done using a Contiki Cooja simulator. The LA-RPL’s performance is compared to RPL, BD-RPL, m-RPL, and A-RPL. However, LA-RPL causes congestion, as it doesn’t consider the trickle timer.

Ainaz Bahramlou and Reza Javidan [21] proposed a data aggregation based RPL (A-RPL) protocol for IoT. In IoT, the routing protocol changes its path frequently due to its resource-constrained nature. It proposed a dynamic method to reconstruct the DODAG quickly, which finds a suitable objective function among the number of objective functions. In this dynamic method, the sink node collects the aggregated data from the downstream nodes. A-RPL selects the parent node based on the environmental changes and control overhead in the network. The performance of A-RPL is compared with RPL and A-RPL. A-RPL provides substantial packet delivery consistency and increases the lifespan of the network. However, it causes congestion in a particular situation, as it does not consider the dynamic trickle timer.

Mauro Conti et al. [22] proposed a reliable group communication protocol (RECOUP) for IoT. RPL is a network routing protocol for IoT. However, it lacks reliability, scalability, and security. So, it proposed a reliable group communication (RECOUP) protocol, which performs cluster-based multicast routing. The performance of RECOUP improves the packet delivery ratio by 25% and decrease the delay by 100 ms. Thus, it extends the network lifetime. However, it takes more energy consumption, as it checks each data packet in each node.

YichaoJin et al. [23] proposed content-centric routing in RPL (CCR-RPL) for IoT. Nowadays, IoT can be used in various applications. However, the routing protocol faces difficulty while collecting the data present in the dense network. To avoid this issue, it proposed a content-centric routing protocol for IoT. It identifies the content via the routing path. Then, the data aggregation process is carried out in the intermediate node from the source to the destination. The simulation has been conducted in COOJA and TelosB. The performance of CCR-RPL is compared to RPL. The CCR-RPL achieves better performance in terms of latency, energy efficiency, and reliability in both the scenarios. However, it creates congestion due to dynamic network conditions.

Ming Zhao et al. [24] suggested a parent cluster RPL (C-RPL) for IoT. C-RPL uses the opportunistic forwarding scheme for synchronization and selects the optimal parent cluster set to reduce the latency between source and destination. While transferring the data, the priority-based scheduling scheme is incorporated in RPL. The simulation has been conducted in NS3. C-RPL performance is compared to RPL and ORPL performance. It achieves not able performance by means of reliability and by avoiding the packet retransmission. However, C-RPL packet loss occurs, as it takes more time to select the cluster parent.

Madan Mohan Agarwal et al. [25] proposed a fuzzy-based data fusion technique for IoT (FLWP). FLWP is proposed to maximize the lifespan of the network. The operations of FLWP is as follows: first, it performs data fusion using fuzzy logic. Later, it predicts the optimal route based on the data fusion value. The performance of FLWP is compared with the AODV routing protocol. It provides superior performance to the AODV protocol. However, it takes a longer time to predict the parent node.

Marc Barcode et al. [26] proposed cooperative interaction in multiple RPL. It proposed cooperative RPL (C-RPL), which creates multiple instances based on the cooperative strategy. The simulation has been conducted in the Contiki Cooja simulator. The C-RPL provides better performance by means of energy consumption and the cost of the nodes in the networks. However, it takes additional time to choose the parent node present in the multiple DODAG.

S. Sankar and P. Srinivasan [27] proposed a fuzzy set based cluster routing (FC-RPL) protocol for IoT. The FC-RPL consists of three phases, namely cluster formation, CH selection, and CH parent selection. The cluster is formed from the Euclidean distance. The CH selection is performed by considering the fuzzy logic over the routing metrics residual energy, centrality, and nearest neighbor node. The CH parent node is selected from the CH parent rank. The simulation has been conducted using the Contiki COOJA simulator. The performance of FC-RPL is compared to RPL. The proposed FC-RPL extends the network lifetime. However, it forms an excess number of clusters in the network.

Yaarob Al-Nidavi et al. [28] proposed a cluster-based routing (MUCBR-RPL) protocol for LLN. The MUCBR protocol divides the entire network into multiple clusters. In each cluster, the MUCBR selects the cluster head based on the residual energy. The CH node collects the data packets from cluster members and performs the data aggregation. Finally, the aggregated data is forwarded to the Sink node. The simulation has been conducted using the COOJA simulator. The performance of MUCBR-RPL provides better performance in terms of network lifetime and reliability. However, initially, it takes time to form the cluster. The literature review of Data aggregation on routing protocols is given in Table 1.

## 3. Network Model

**Figure 1:**
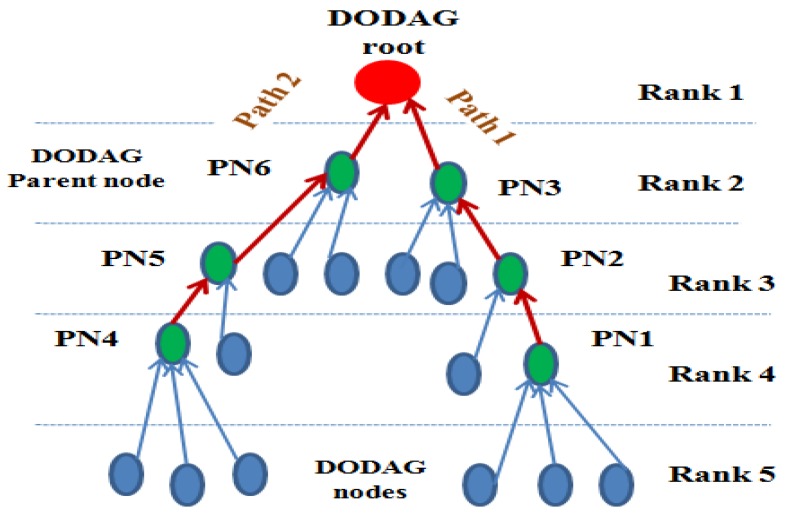
Energy and Delay Aware Data aggregation in Routing Protocol (EDADA-RPL) Network Model.

The IoT network consists of ‘N’ number of nodes and one DODAG root. The nodes are randomly deployed in the network. The EDADA-RPL follows the tree-based approach. The sensor node generates the data and transmits it to the parent node. Each parent node collects the data packets and performs the data aggregation using compressed sensing (CS) theory [29]. Likewise, the data aggregation operation performs from a downward parent to the root of the DODAG. Finally, the DODAG root obtains the compressed data and it performs the data recovery using the matching pursuit algorithm. Figure 1 shows the network model of EDADA-RPL.

**Figure 2:**
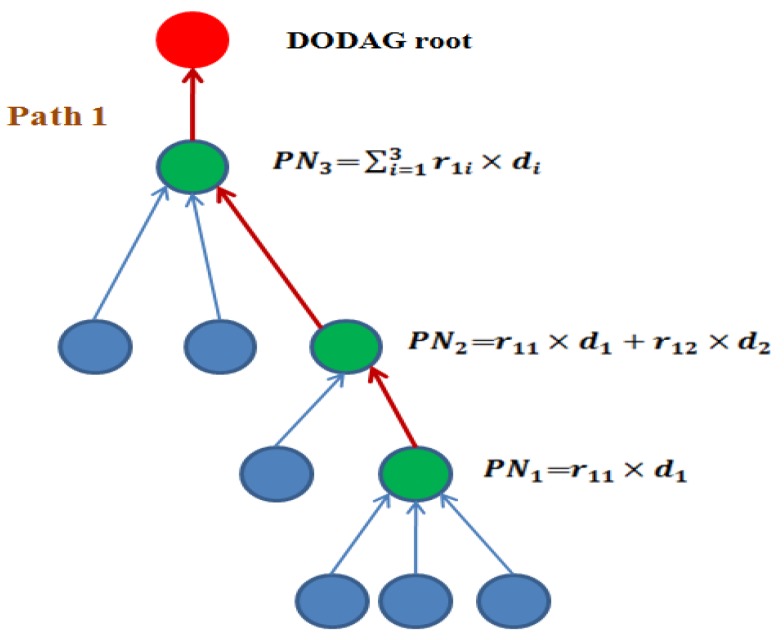
Data collection and aggregation from node PN1 to Destination Oriented Directed Acyclic Graph (DODAG) root using compressed sensing (CS) theory.

The Figure 2 shows the data collection and aggregation process using CS theory. In this, the path1 contains the subset of nodes in the DODAG, which are represented the data aggregation process. The parent node (PN) generates the aggregated data $d_{i}$ multiplied by the random weight value $r_{i}$. In each level in DODAG, the current parent node (PN) obtains the aggregated data in their child nodes along with downward parent transmitted data $PN_{k}=\overset{k}{\underset{i=1}{\sum }}r_{1i}\times d_{i}$. The detailed discussions of the data aggregation process are given in Section 5.

Assumptions:

- Nodes are deployed randomly on the network.
- All nodes are having the same energy.
- The rate of data transfer is one packet a minute.

## 4. Data Aggregation Using Compressed Sensing Theory

### 4.1. Data Aggregation

**Figure 3:**
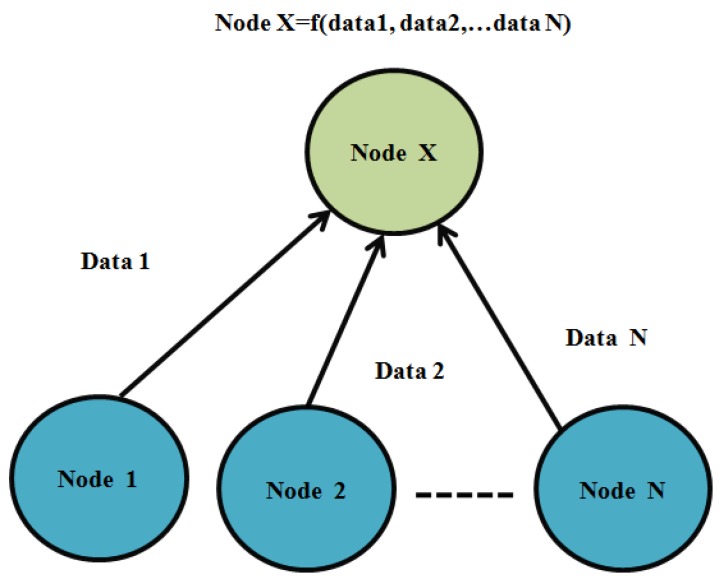
Data aggregation process.

Data aggregation is an essential process in wireless routing for collecting the data from various sources in the network [15]. It aggregates the sensor’s data, which removes the data redundancy and reduces the number of data transmissions. Thus, it saves the energy on the network nodes in IoT. Data fusion clubs the sensor’s data and removes the noise from various sources. Finally, it generates accurate data for data transmission. The data aggregation process is shown in Figure 3.

### 4.2. Compressed Sensing Theory

**Figure 4:**
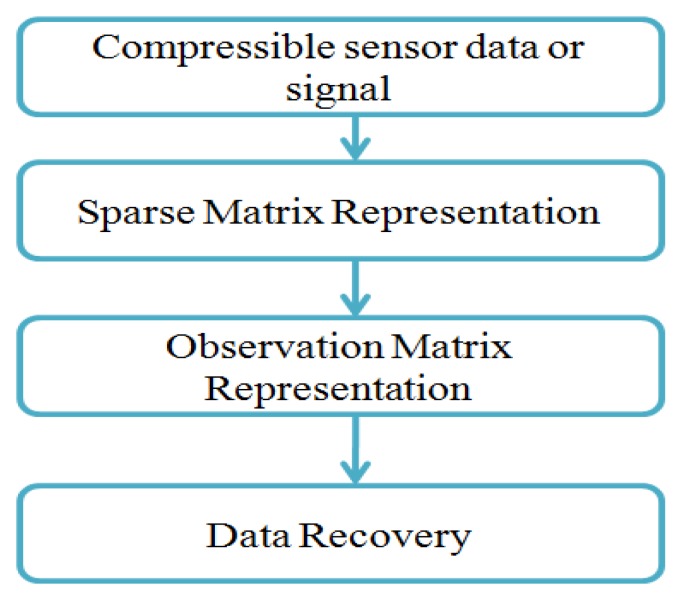
Compressed sensing theory.

A sensor data or signal can be converted from lower dimensional space to higher-dimensional space is called sparse. Generally, the data is converted into a sparse matrix. Later, it converts the sparse matrix into the observation matrix. Finally, the data recoveries will perform from the observation matrix [30]. Figure 4 shows the overall process of the compressed sensing theory.

#### 4.2.1. Sensor Data

In IoT, the nodes are deployed randomly in the network. Each sensor nodes generate the data N $\times 1\;matrices\;d=\left[\begin{array}{c} d_{1}\\ d_{2}\\ .\\ .\\ d_{n} \end{array}\right]$ and it can be represented as d = ${\left\{d_{1},\;d_{2},\;d_{3}\ldots ..d_{n}\right\}}^{T}$.

#### 4.2.2. Sparse Matrix Representation

A sparse matrix is matrix representation which contains the sensor data d of the network nodes N. It is an orthogonal domain representation [31]. The data aggregation operation performs a regular interval, ‘t’. The sparse matrix at parent node ‘s’ representation is given in Equation (1).

(1) $$s=r_{i,t}\times d_{i,t}$$

where $r_{i,t}$ = {$r_{1,t},r_{2,t},\ldots r_{i,t}\}$, $r_{i,t}$ is a random number and $d_{i,t}$ is a sensor data of respective time interval.

#### 4.2.3. Observation Matrix Representation

The DODAG root collects the aggregated data from each path i = 1,2,…,M. The DODAG root collects data in path 1, represented in Equation (2).

(2) $$y_{1}=\overset{k}{\underset{i=1}{\sum }}r_{1i}\times d_{i}$$

Similarly, the DODAG root collects the aggregated data from the M paths, represented in Equation (3).

(3) $$y=\overset{M}{\underset{i=1}{\sum }}y_{i}$$

where $y_{i}$ indicates the aggregated data in a particular path and y indicates the aggregated data from the M paths.

#### 4.2.4. Data Recovery

The data can be recovered through solving the combinatorial problem, represented in Equation (4).

(4) $$\min_{{d\varepsilon R}^{N}}\|d\|\;\mathrm{such}\;\mathrm{that}\;y=r\;\times \;d$$

## 5. Energy and Delay Aware Data Aggregation in RPL

The Proposed Energy and Delay Aware Data aggregation in RPL (EDADA-RPL) has two processes, namely parent selection and data aggregation. The process of parent selection uses routing metric residual energy (RER) to choose the best possible parent for data transmission. The data aggregation process uses the compressed sensing (CS) theory in the parental node to combine data packets from the child nodes. Finally, the aggregated data transmits from a downward parent to the DODAG root or sink. The DODAG root gathers aggregated data and conducts the reconciliation process to recover the original data.

### 5.1. Parent Selection

In EDADA-RPL, the DODAG root node broadcasts the DODAG Information Object (DIO) to the neighbor nodes in the network. The DODAG Advertisement Object (DAO) message is sent to the parent or DODAG root by the participant node. The DODAG root or parent node sends the signal of the DODAG Advertisement Object-Acknowledgement (DAO-ACK) to the child node within the trickle interval. The participant node chooses the parent node depended on the DODAG node-level routing remaining metric energy (RER).

Residual energy shows the present energy available in the RPL routers. The RER measures the difference between the original energy and the energy currently consumed by the node [32]. The formula for calculating the remaining energy is provided in Equation (5).

(5) $$\mathrm{RER}\left(\mathrm{Ni}\right)=\frac{E_{\mathrm{Initial}}-E_{\mathrm{Depleted}}}{E_{\mathrm{Initial}}}$$

### 5.2. Parent Rank Calculation

Rank indicates the root of the DODAG, how far from the node of the participant. The node ‘x’ rank computes from the parent node(x) rank and its value, Rank_Increase_Value. The value of Rank Increase calculates the value of residual energy and the value of MinHop_RankIncrease. The MinHop_RankIncrease value is 256, which is the default value in the rank calculation [33,34]. The calculation of the rank is shown in Equations (6) and (7).

(6) Rank(x)=Rank(parentNode(x))+Rank_Increase_Value

(7) Rank_Increase_Value=RER+MinHop_RankIncrease

The EDADA-RPL parent selection algorithm is given in Algorithm 1.
**Algorithm 1: EDADA-RPL parent selection**Input: DIO, DAO, DAO-ACK, DIO_REROutput: Optimal Parent1:**For** preferred_ParentNodeparentNode-list **do**2: compute RER   $\mathrm{RER}\left(\mathrm{Ni}\right)=\frac{E_{\mathrm{Initial}}-E_{\mathrm{Depleted}}}{E_{\mathrm{Initial}}}$3:compute the Rank(N)      
Rank(x)=Rank(parentNode(x))+Rank_Increase_Value
4:Calculate the Rank_Increase_ValueRank_Increase_value=RER+MinHop_RankIncrease5:**If**Best_ParentNode>= Preferred_ParentNode**Then**Best_ParentNode=Preferred_ParentNode6:**End**7:**While**preferred_ParentNode==Best_ParentNode**do**SourceNode=Preferred_ParentNode8:**End**9:**End**10:Return Optimal Parent

### 5.3. Data Aggregation in Parent Node Using CS Theory

In the data aggregation process, the sensor nodes transmit the data $d={\left\{d1,d2\ldots dn\right\}}^{T}$ to the parent node. The parent node $PN_{1}$ performs the data collection and aggregation operation and it transmits the aggregated data packets to the DODAG root [35]. Each parent node gets the weighted sum of the random number multiply by the sensor data ‘*d*’, called a sparse matrix. In path 1, the node $PN_{1}$ generates the values are $r_{11}\times d_{1}$, given in Equation (8).

(8) $$PN_{1}=r_{11}\times d_{1}$$

where $r_{11}$ indicates the random values of parent node 1 ($PN_{1}$) and $d_{1}$ indicates the aggregated data of $PN_{1}$.

First, the parent node $PN_{1}$ sends the aggregated data $r_{11}\times d_{1}$ to $PN_{2}$. Second, the parent node $PN_{2}$ collects the data from $PN_{1}$ and also it collects and aggregates the data from its child nodes. The mathematical representation is given in Equation (9).

(9) $$PN_{2}=r_{11}\times d_{1}+r_{12}\times d_{2}$$

where $r_{11}\times d_{1}$ indicates the aggregated data in $PN_{1}$ and $r_{12}\times d_{2}$ indicates the aggregated data in $PN_{2}$.

Third, the parent node $PN_{2}$ sends the aggregated data to node $PN_{3}$. The node $PN_{3}$ performs the data aggregation operation, given in Equation (10).

(10) $$PN_{3}=\overset{k}{\underset{i=1}{\sum }}r_{1i}\times d_{i}$$

where k indicates the number of parent nodes in each path, $r_{1i}$ indicates random number of each parent node in path 1.

In path 1, the parent nodes transmit the data from $PN_{1}$ to DODAG root. The DODAG root collects the aggregated data in path 1 is called the observation matrix and it is given in Equation (11).

(11) $$y_{1}=\overset{k}{\underset{i=1}{\sum }}r_{1i}\times d_{i}$$

where $y_{1}$ indicates the DODAG root collects the aggregated data in path 1.

Similarly, the DODAG root collects the aggregated data from M paths and the DODAG root receives the data packets $y_{i}$, where i = 1,2,…,M. So, we can represent the data aggregation process mathematically, given in Equation (12).

(12) $$\left[\begin{array}{c} y_{1}\\ y_{2}\\ .\\ .\\ y_{M} \end{array}\right]=\left[\begin{array}{ccccc} r_{11} & r_{12} & .. & .. & r_{1N}\\ r_{21} & r_{22} & .. & .. & r_{2N}\\ . & . & .. & .. & .\\ . & . & .. & .. & .\\ r_{M1} & r_{M2} & .. & .. & r_{MN} \end{array}\right]\left[\begin{array}{c} d_{1}\\ d_{2}\\ .\\ .\\ d_{N} \end{array}\right]$$

Finally, we can reconstruct the weighted sum of aggregated data from the M path to N node’s original data using CS theory. Thus, we can reduce the number of data transmission (M<N) in the network.
**Algorithm 2: Data Aggregation Using Compressed Sensing Theory****Input:** Sensor data d = {d1, d2,…, dn}**Output:** Compressed data y1:Compute the data aggregation fromPN1 to DODAG root $y_{1}=\overset{k}{\underset{i=1}{\sum }}r_{1i}\times d_{i}$

2:Compute the data aggregation in DODAG root from the M paths  $\left[\begin{array}{c} y_{1}\\ y_{2}\\ .\\ .\\ y_{M} \end{array}\right]=\left[\begin{array}{ccccc} r_{11} & r_{12} & .. & .. & r_{1N}\\ r_{21} & r_{22} & .. & .. & r_{2N}\\ . & . & .. & .. & .\\ . & . & .. & .. & .\\ r_{M1} & r_{M2} & .. & .. & r_{MN} \end{array}\right]\left[\begin{array}{c} d_{1}\\ d_{2}\\ .\\ .\\ d_{N} \end{array}\right]$
3: Return the aggregated data y.

### 5.4. Data Reconstruction Using Matching Pursuit Algorithm

Data reconstruction is a recovery technique, which is used to recover the compressed data over the network nodes. The DODAG root receives the compressed data in the form of observation matrix from M paths. Also, the DODAG root recovers the sparse matrix accurately using matching pursuit algorithm. Finally, the DODAG root rebuilds the compressed data and it matches with original data [36].

In matching pursuit algorithm, the EDADA-RPL recovers the data d from the input y=r×d. The residual error ($r_{error}$) is initialized as y. The sparse matrix in each parent node PN is generated and it is given in Equation (13)

(13) $$PN=\bar{d_{i-1}}+r^{T}\times r_{error}$$

where $r^{T}$ indicates the random number in each parent node (PN).

The data recovery process will be continued until both original and recovered data are the same. The pseudo-code of the matching pursuit algorithm is given in Algorithm 3.
**Algorithm 3: Data Reconstruction Using Matching Pursuit Algorithm****Input:** compressed data y=r×d**Output:** Reconstructed data $\bar{d}$1:Initialize: $\bar{d_{0}}$ = 0, $r_{error}=0$ and i = 0 2:iteration:3:  i = i + 14:  $PN=\bar{d_{i-1}}+r^{T}\times r_{error}$
5:  $\bar{d_{i}}=argmax_{i=\left\{1,2,..N\right\}}\left|PN\right|$
6:Compute residual error $r_{error}=y-r\times \bar{d_{i}}$
7:**If** there is no error in $r_{error}$**then**8:**return d**9:**End**10:**End**

We perform the reconstruction at the DODAG root and receive the original data d of N nodes. Finally, we compute the mean square error and its value is very low. So, it proves that EDADA-RPL provides less data loss when compared to other routing protocols.

## 6. Result and Discussions

In our simulation, all the nodes have equal energy, which is deployed randomly in the network. The network contains 120 RPL routers and one DODAG root. The data transfer interval is 60 s. We have taken the sky mote for our simulations [37]. The simulation has been conducted in the COOJA simulator [38]. The data transfer rate is one packet per minute. All results presented in the figures below are averaged over 10 simulation runs and error bars show the 95% confidence intervals. The simulation parameters are given in the Table 2.

### 6.1. Performance Evaluation Metrics

The performance of proposed EDADA-RPL is compared with the familiar existing protocols RPL and LA-RPL.

**Packet delivery ratio:** the proportion of data packets received successfully to the total sent.

**Delay:** time taken for transmitting the data from source to DODAG root.

**Energy consumption**: the number of miliwatts spent on transmitting data packets in the network from source to DODAG root.

**Packet overhead:** the amount of control packets generated in the transmission of data packets.

### 6.2. Simulation Results

#### 6.2.1. End-to-End Delay

**Figure 5:**
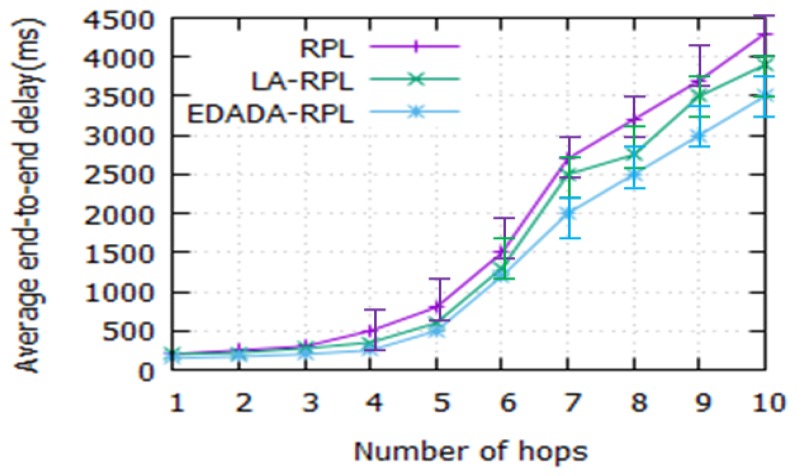
Average end-to-end delay vs. number of hops.

Figure 5 shows the average end-to-end delay between the participant and the root of DODAG for a number of hops. In general, the LLN network takes more time to transfer the data, due to its resource-constrained nature. The effective routing mechanism tackles these issues and increases the network performances. However, the end to end delay is one factor to deliver the sensor information on time to the sink time. It is noted that EDADA-RPL, LA-RPL, and RPL delays are 3.5 s, 3.9 s, and 4.2 s respectively, when the hop count number is 10. It is also observed that the delay increases as the number of hop count increases. It is due to a reduction in path breakage and the amount of redundant data transmission.

#### 6.2.2. Number of Parent Change

**Figure 6:**
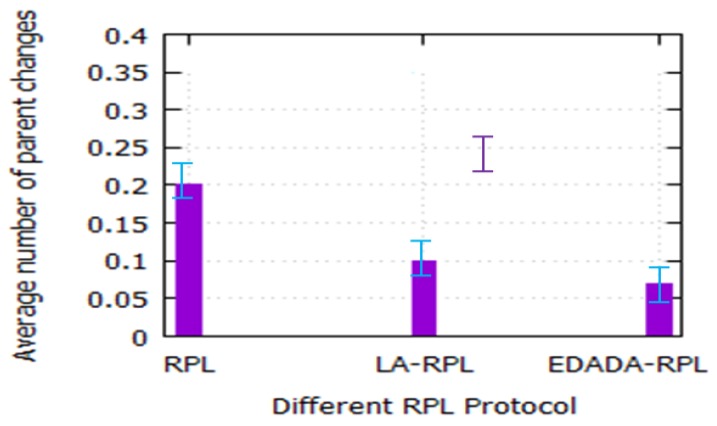
Average number of parent change vs. various RPLs.

Figure 6 depicts the number of parent changes over time. The parent change values indicate network stability. If the network has fewer parent changes during the simulation, it can have more stability and also the chance of packet losses is very low. The parent change values of EDADA-RPL, LA-RPL, and RPL for a 1-h simulation are 0.07, 0.10, and 0.20, respectively. The proposed protocol is introduced the compressed sensing theory to aggregate the data from its child node. It is a hybrid aggregation technique, which reduces the data losses from the source to the destination. Thus, it reduces the number of redundant data transmission and path breakages. So, it maintains route stability during the data transmission.

#### 6.2.3. Number of Hop Count

**Figure 7:**
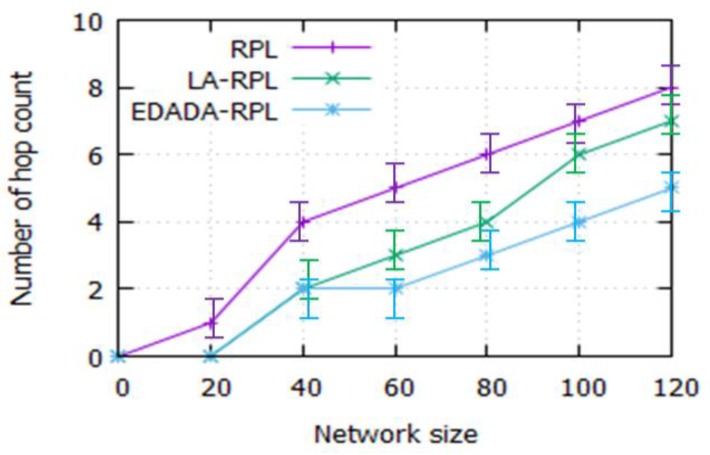
Number of hop count vs. Network size.

Figure 7 depicts the number of hop count for the network size. In a dense network, the number of hops between the sources to the destination is very high. The reason is that the numbers of nodes are deployed randomly in a small network area. The simulation experiment is conducted and the results are noted for the performance analysis. The hop counts of RPL, LA-RPL and EDADA-RPL are 8, 7, and 5 respectively, for the network size of 120. It is also found that the size of the network is increasing; the number of hop counts also increases. In EDADA-RPL, the number of hop count decreases, as it performs the data aggregation during the data transmission. Thus, it reduces the number of hop count and provides route stability among the nodes in the network.

#### 6.2.4. Packet Loss Ratio with Presence of Failed Node Scenario

**Figure 8:**
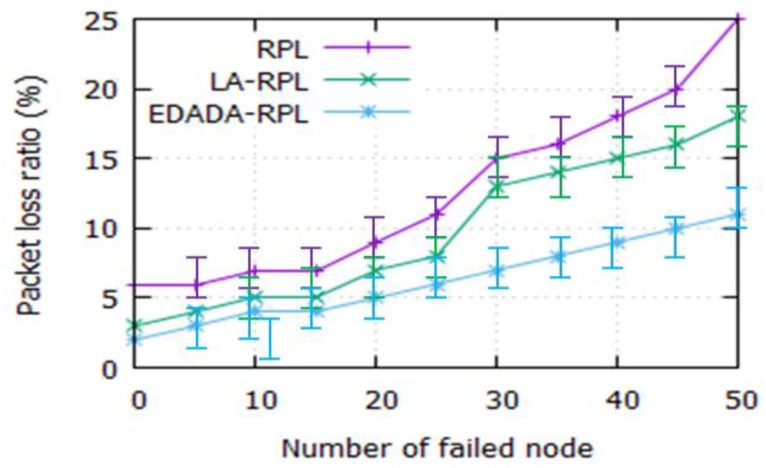
Packet loss ratio vs. number of failed nodes.

Figure 8 depicts the packet loss ratio for the failed node scenario. It is noted that RPL, LA-RPL and EDADA-RPL’s packet loss ratiosare 25%, 18%, and 12%, respectively, while the node size failed is 50. It is also found that, as the number of failed node size decreases, the packet loss increases.It is due to the lack of neighbor nodes, coverage issues, and difficultyin finding alternate nodes. Nevertheless, the EDADA-RPL identifies an alternative node to immediately transfer data to the DODAG root.

#### 6.2.5. Actual and Reconstructed Sensor Data

**Figure 9:**
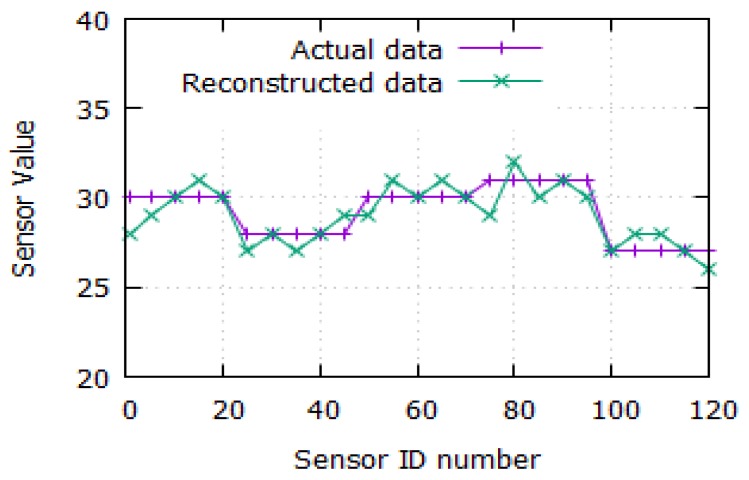
Sensor value vs. sensor ID number.

Figure 9 depicts the actual data for the reconstructed sensor data. It is observed that the actual sensor data and reconstructed sensor data in EDADA-RPL is almost similar when compared to both the values. It is noted that the sensor IDs are represented from 1 to 120. Here, the sensor readings are nearly 30. The sensor values are varying, due to various reasons such as noise, the distance between the source and the destination, etc. So, the sensor values are falling up and down, but it maintains the consistency value by using compressed sensing theory. Hence, the EDADA-RPL recovers the original data from the source node without any losses.

#### 6.2.6. Node Energy Consumption

**Figure 10:**
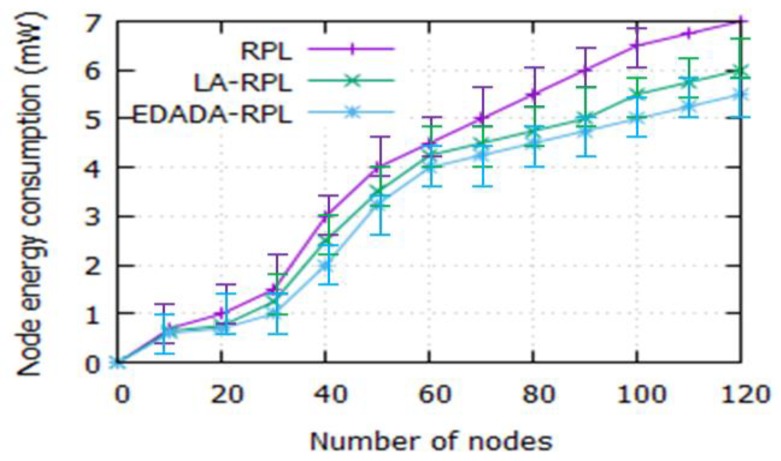
Node energy consumption vs. number of nodes.

Figure 10 illustrates the amount of node energy consumption in the network. Energy is a scarce resource in IoT, as it contains the resource-constrained devices. It is noted that the energy consumption of RPL, LA-RPL, and EDADA-RPL are 7 mW, 6 mW, and 5.5 mW, respectively, for a network size of 120. The proposed EDADA-RPL reduces the energy consumption by 1.5 mW. It is also found that the node energy consumption increases, as the number of nodes in the network increases. It is due to the number of nodes required to sense, listen, accumulate, and transmit the data from one node to another. Thus, it increases the energy consumption among the nodes in the networks. However, the EDADA-RPL consumes less energy when compared to RPL and LA-RPL. The reason is that it reduces the number of redundant data in the network from one node to another.

#### 6.2.7. Reconstruction MSE Value

**Figure 11:**
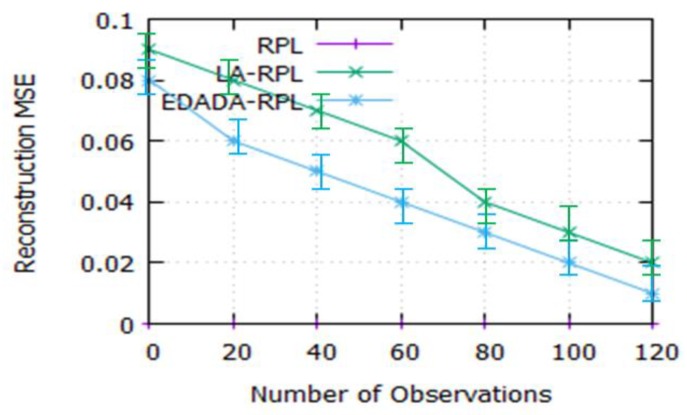
Number of observations vs. reconstruction MSE.

Figure 11 depicts the reconstruction of Mean Square Error (MSE) value for the number of observations. It is noted that reconstruction MSE of LA-RPL and EDADA-RPL are 0.02 and 0.01 respectively for the 120th iterations. In that, the RPL always the MSE values are 0 because there is no compression process during the data transmission. Reconstruction of the MSE value has been observed to decrease as the number of observations increases.It is due to the variation in node energy and data traffic between the nodes of the network.

#### 6.2.8. Packet Loss Ratio

**Figure 12:**
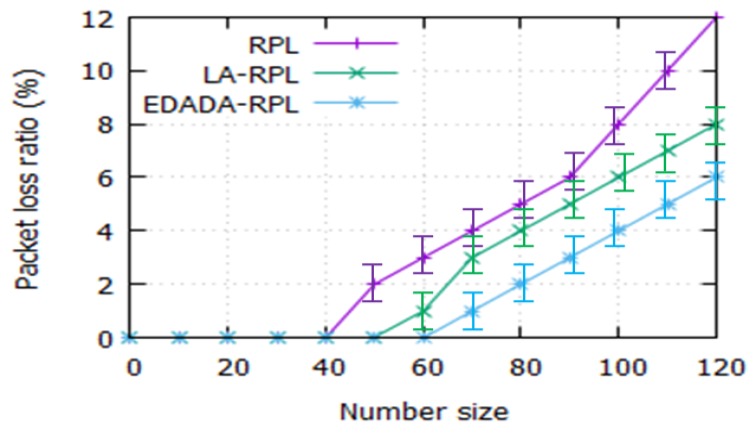
Packet loss ratio vs. network size.

Figure 12 depicts the packet loss ratio with respect to the network size. In our simulation, we observed that packet loss ration increases dramatically, while increasing the network size. It is noted that the RPL, LA-RPL, and EDADA-RPL packet loss ratios are 12%, 8%, and 6% respectively, for a network size of 120. It confirms that the data packet loss is also found to increase, as the network size increases. It is due to the lack of neighbor nodes, coverage issues and difficult to find alternate nodes. However, EDADA-RPL finds an alternate node to transfer the data quickly to the DODAG root.

## 7. Conclusions and Future Works

Energy conservation is important role in Internet of Things (IoT). Therefore, routing plays an essential role in IoT. We proposed Energy and Delay Aware Data aggregation in Routing Protocol (EDADA-RPL) for IoT. It has two processes, namely parent selection and data aggregation. The parent selection process uses the routing metric residual energy (RER) to select the optimal parent for data transfer. The data aggregation process uses the compressed sensing (CS) theory in the parent node to combine data packets from the child nodes. Then, the aggregated data is transmitted to the DODAG root from the downward parent. The DODAG root node collects all the aggregated data and performs the reconstruction operation to get original data. The performance of EDADA-RPL is compared with RPL and LA-RPL. The EDADA-RPL decreases the delay and packet loss ratio by 8–15 s and 6–10%, respectively.

In our future work, we plan to deploy sky motes in a real-time environment and toanalyse EDADA-RPL efficiency in various scenarios.

## Figures and Tables

**Table 1:** Literature review of Data aggregation on Routing Protocol for Low Power and Lossy Networks (RPL).

S.No	Protocol	Author’s	Proposed Technique	Improvement	Limitations
1	LA-RPL	Mohammad HosseinHomaei et al.	learning automata-based dynamic data aggregation	Extends the network lifetime	It does not consider the trickle timer
2	A-RPL	Ainaz Bahramlou and Reza Javidan	data aggregation based RPL	Increased the network lifetime	congestion occurs in a particular situation
3	RECOUP-RPL	Mauro Conti et al.	cluster-based multicast routing	Increased the packet delivery ratio	It takes more energy consumption, as it checks each data packets in each node.
4	CCR-RPL	YichaoJin et al.	content-centric routing	better performance in terms of latency, energy efficiency and reliability	Create the congestion due to dynamic network conditions.
5	C-RPL	Ming Zhao et al.	Cluster parent routing	Increased the reliability	It takes more time to choose the cluster parent
6	FLWP	Madan Mohan Agarwal et al.	fuzzy-based data fusion technique	It provides superior performance than the AODV	It takes a longer time to predict the parent node
7	C-RPL	Marc Barcode et al.	cooperative interaction	Increased the network lifetime	It takes additional time to choose the parent node present in the multiple DODAG
8	FC-RPL	S. Sankar and P. Srinivasan	cluster routing	Extended the network lifetime	It forms the more number of clusters in the network.
9	MUCBR-RPL	Yaarob Al-Nidavi et al.	cluster routing	Improved the network lifetime and packet delivery ratio	Initially, it takes time to form the cluster.

**Table 2:** Simulation parameters.

Parameter	Value
Operating System	Contiki 2.7
Simulator	COOJA
Initial Energy	1500 mA
Routing Protocol	RPL
Simulation Time	1 h
Network area	300 m × 300 m
Topology	Random
Node Type	Skymote
Number of Nodes	120
MAC Layer	802.15.4
Data Transmission Interval	60 sec
Physical Layer	Two Ray Ground Propagation Model
RPL Parameter	MinHopRankIncrease = 256
